# The Risk of Road Traffic Injuries Caused Hospitalization and the Risk of Mental Health Illness: A Nationwide, Matched‐Cohort, Population‐Based Study in Taiwan

**DOI:** 10.1002/brb3.70993

**Published:** 2025-11-10

**Authors:** Yu‐An Chen, Wu‐Chien Chien, Li‑Yun Fann, Ying‐Che Huang, Chi‐Hsiang Chung, Tsu‐Hsuan Weng, Chun‐Teng Tsai

**Affiliations:** ^1^ Department of General Medicine, Tri‐Service General Hospital National Defense Medical University Taipei City Taiwan Republic of China; ^2^ Department of Medical Research, Tri‐Service General Hospital National Defense Medical University Taipei City Taiwan Republic of China; ^3^ Graduate Institute of Life Sciences, College of Biomedical Sciences National Defense Medical University Taipei City Taiwan Republic of China; ^4^ Graduate Institute of Public Health, College of Public Health National Defense Medical University Taipei City Taiwan Republic of China; ^5^ Graduate Institute of Medical Sciences College of Medicine National Defense Medical University Taipei City Taiwan Republic of China; ^6^ Taiwanese Injury Prevention and Safety Promotion Association Taipei City Taiwan Republic of China; ^7^ Tzu Chi University College of Nursing Hualien City Hualien County Taiwan Republic of China; ^8^ Department of Anesthesiology, Yangming Branch Taipei City Hospital Taipei City Taiwan Republic of China

## Abstract

**Background:**

While road traffic injuries (RTIs) are a significant global health concern, medical attention has predominantly addressed physical injuries, often overlooking the psychological impact on survivors. The mental health consequences of such traumatic events remain understudied and inadequately addressed in clinical practice.

**Aims:**

This study examined the association between RTIs and subsequent mental health disorders.

**Methods and Procedures:**

We conducted a retrospective cohort analysis using claims data from Taiwan's National Health Insurance Research Database (NHIRD) between 2000 and 2015. The exposed cohort comprised 39,870 patients hospitalized following RTIs. These individuals were matched 1:27 with 1,076,911 control patients hospitalized for non‐RTI‐related conditions, using propensity score matching based on sex, age, and admission date. Multivariable Cox proportional hazards regression models were employed to compute adjusted hazard ratios (aHRs), assessing the risk of psychiatric diagnoses in the RTI group compared to controls.

**Outcomes and Results:**

Patients hospitalized following RTIs demonstrated a substantially increased risk of subsequent mental health disorders compared to matched controls (adjusted hazard ratio [aHR] = 2.20, 95% confidence interval [CI]: 1.66‐2.79). This elevated risk remained statistically significant after comprehensive adjustment for potential confounding variables.

## Graphical abstract

This nationwide matched‐cohort study using Taiwan's National Health Insurance Research Database (NHIRD) examined the long‐term mental health outcomes following hospitalization due to road traffic injuries. Patients with road traffic injury (RTI) ‐related hospitalizations had a significantly higher risk of developing depression, anxiety, and post‐traumatic stress disorder compared with matched controls. Early psychiatric screening and timely interventions should be considered in trauma care and health policy planning.

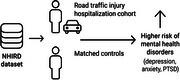

## Abstract (es)

What does this paper add?

This nationwide, matched‐cohort, population‐based study in Taiwan showed that people with hospitalization due to RTIs are associated with increased risk of overall mental health issues.

## 1 Introduction

Road traffic injuries (RTIs) represent a critical global health challenge, accounting for substantial morbidity, mortality, and long‐term disability. These incidents encompass diverse injury patterns ranging from traumatic brain and spinal cord injuries to complex polytrauma involving fractures and internal organ damage. The World Health Organization's 2023 report highlights the alarming scale of this issue, with road traffic accidents claiming approximately 1.19 million lives annually. Liu et al. (2022) analyzed mortality causes in 5–19‐year‐olds from 2000 to 2019 and confirmed RTIs as the leading cause globally in the 5–19 range, particularly in strong males.

While the physical consequences of motor vehicle accidents have been well‐documented, the psychological impact on survivors remains insufficiently explored. Current research has predominantly focused on mental health outcomes following specific neurological injuries, particularly traumatic brain and spinal cord injuries, creating a significant knowledge gap regarding the broader population of motor vehicle accident survivors.

To address this gap, we conducted a nationwide cohort study utilizing Taiwan's National Health Insurance Research Database (NHIRD). Our investigation specifically examines the risk of mental health disorders among patients hospitalized following motor vehicle collisions, providing comprehensive population‐level evidence on this understudied aspect of post‐accident care.

## 2 Methods

### 2.1 Data Source

We conducted a retrospective cohort study using Taiwan's Longitudinal Health Insurance Database (LHID), a nationally representative subset of the National Health Insurance Research Database (NHIRD). Our analysis spanned 15 years (2000–2015) and included both inpatient and outpatient records to examine mental health outcomes following hospitalization for motor vehicle accidents.

Taiwan's compulsory National Health Insurance (NHI) program, implemented in 1995, achieved near‐universal coverage by 2009, enrolling 97% of healthcare providers and 99% of the population (23 million residents). The database employs ICD‐9‐CM codes (Chinese Hospital Association, 2000) for disease classification, with psychiatric diagnoses including mood disorders, anxiety disorders, post‐traumatic stress disorder (PTSD), substance use disorders, and psychotic disorders confirmed by board‐certified psychiatrists or neurologists.

The NHI Administration ensures data quality through random audits (1% of outpatient and 5% of inpatient records), with multiple validation studies confirming diagnostic accuracy.

### 2.2 Study Design and Sampled Participants

#### 2.2.1 Study Design

This study was of a retrospective matched‐cohort design.

#### 2.2.2 Sample

**FIGURE 1:**
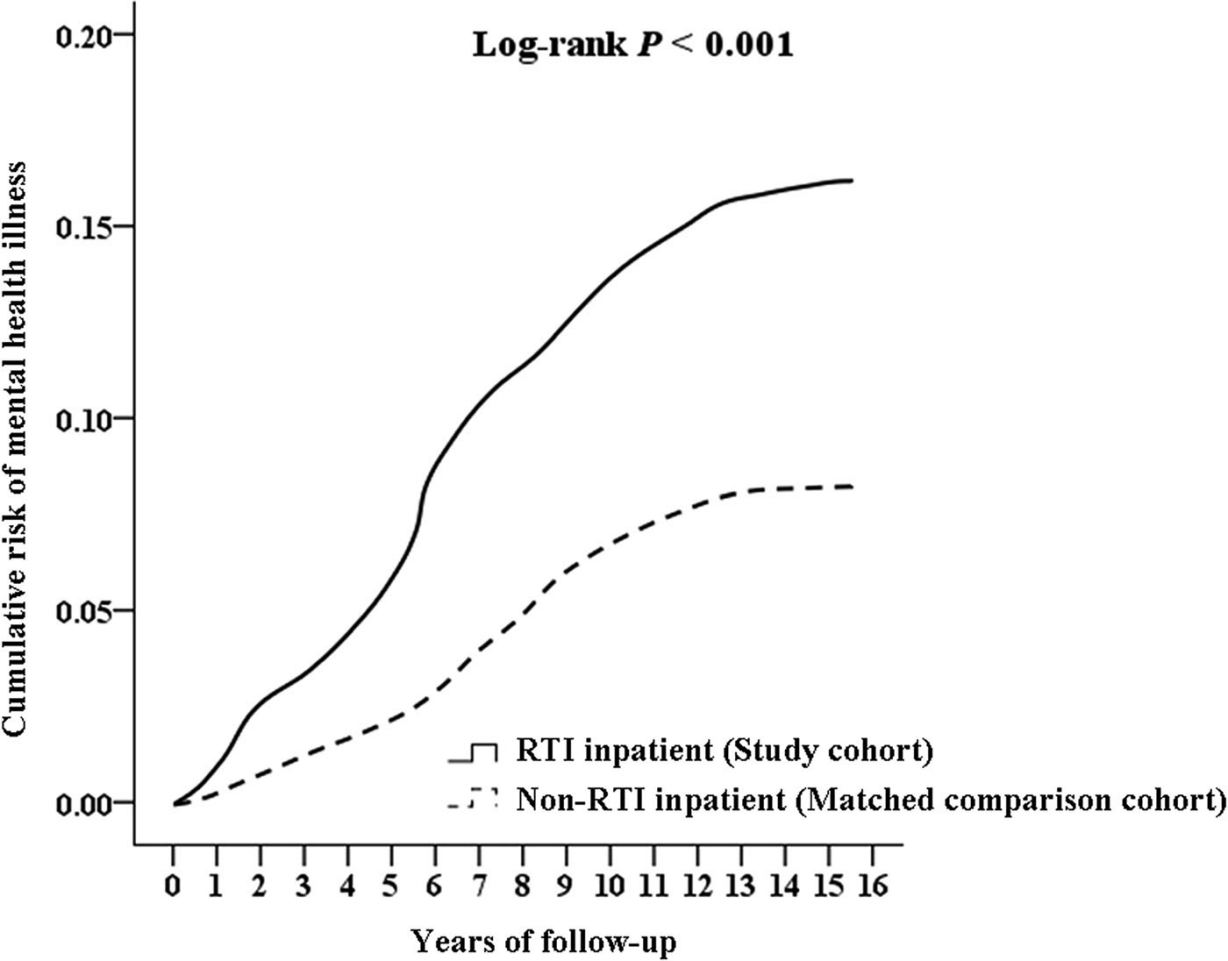
Kaplan–Meier survival curves for RTI inpatients and matched non‐RTI controls.

This retrospective cohort study identified patients hospitalized for road traffic injuries (RTIs) between January 1, 2000, and December 31, 2015, through ICD‐9‐CM diagnostic codes (E810‐E819), including all relevant subcategories for different accident types and involved parties. The specific code definitions are detailed in Supplementary Table S1. The analysis was restricted to patients requiring at least 24 h of hospitalization and excluded those with documented injuries prior to 2000, pre‐existing mental health conditions, missing follow‐up data, or unspecified gender. From an initial pool of 19,936,512 eligible patients, we identified 47,161 RTI cases who were then matched with 18,893,351 control patients (1:400 ratio) hospitalized for non‐RTI reasons using propensity scores that accounted for demographic characteristics (age, sex, income), clinical factors (comorbidity profile), healthcare system variables (hospital accreditation level), and environmental determinants (urbanization level of residence, admission season) (Figure 1).

#### 2.2.3 Covariates

The analysis adjusted for multiple potential confounding variables across several domains. Demographic covariates included sex, age group (< 5, 5–14, 15–24, 25–44, 45–64, and ≥ 65 years), and monthly income categorized into three levels (< 18,000, 18,000‐34,999, and ≥ 35,000 NTD). Clinical factors comprised hospitalization outcome (survival or death) and presence of comorbidities. Healthcare system variables included hospital accreditation level as determined by the Taiwan Joint Commission on Hospital Accreditation, which evaluates institutions based on size, facilities, staff qualifications, and service scope. Geographic characteristics were accounted for through urbanization levels (Liu et al., 2022, 1–4) of patients' residential areas, classified according to population size and socioeconomic indicators. Specifically, level 1 represented major metropolitan centers with populations exceeding 1.25 million and significant political‐economic importance; level 2 included regional cities (500,000–1,249,999 inhabitants); while levels 3 and 4 encompassed midsize towns (150,000–499,999) and rural areas (< 150,000), respectively. Seasonal variations were controlled for through inclusion of hospitalization season in the analysis.

#### 2.2.4 Comorbidities

Baseline comorbidities were identified using ICD‐9‐CM codes and included the presence of catastrophic illness as defined by the Ministry of Health and Welfare, as well as specific conditions such as diabetes mellitus (ICD‐9‐CM code 250), hypertension (codes 401–405), hyperlipidemia (code 272), myocardial infarction (codes 410 and 412), and cardiovascular disease (codes 480–489). Additional comorbidities included chronic obstructive pulmonary disease (COPD; codes 490–496), pneumonia (codes 480–488 and 507), respiratory failure (code 518.8), kidney disease (codes 580–589), and epilepsy (codes 345 and 780.3).

These comorbidities were chosen because they are prevalent in the Taiwanese population, have well‐defined and reliable ICD‐9‐CM coding within the NHIRD, and are recognized priorities in Taiwan's public health policy. Including them as baseline covariates allowed for appropriate adjustment of potential confounding effects, thereby isolating the association between RTI–related hospitalization and subsequent mental health outcomes.

#### 2.2.5 Outcome Measures

Participants were prospectively followed from their index hospitalization date until the first occurrence of any mental health diagnosis, with outcomes ascertained through comprehensive psychiatric evaluations. Mental health disorders (for definitions, see Supplementary Table S1) were identified using standardized ICD‐9‐CM codes and required either at least three outpatient or emergency department visits with consistent diagnoses from board‐certified psychiatrists or neurologists or any psychiatric hospitalization. The assessed conditions included mood disorders (depression [296.2–296.3, 300.4, 311], bipolar disorder [296.0, 296.4–296.8]), anxiety disorders (300), trauma‐related disorders (acute stress disorder [308] and post‐traumatic stress disorder [309.81]), substance use disorders (291–292, 303–305), psychotic disorders (schizophrenia [295]), neurocognitive disorders (dementia [290, 331.0]), and other behavioral conditions (sleep disorders [307.4, 780.5], personality disorders [301], and behavioral disorders [312]).

### 2.3 Data Analysis

All analyses were performed using IBM SPSS Statistics (version 22.0; IBM Corp., Armonk, NY). Baseline characteristics were compared using *χ^2^* tests for categorical variables and independent *t*‐tests for continuous measures. The primary analysis employed multivariate Cox proportional hazards regression to estimate injury risk, with results reported as adjusted hazard ratios (aHRs) and 95% confidence intervals. Survival analysis was conducted using Kaplan–Meier curves with log‐rank testing to compare cumulative incidence between groups. All tests were two‐tailed, with statistical significance set at *α* = 0.05.

We also performed sensitivity analyses to test the robustness of our findings, which included analysis by mental health illness subtype, exclusion of events occurring within the first year of follow‐up, and exclusion of events occurring within the first five years of follow‐up. The results of these analyses are presented in Supplementary Table S6.

## 3 Results

### 3.1 Sample Characteristics

The study cohort comprised 47,125 patients hospitalized for road traffic injuries (RTIs), with a predominance of male participants (57.95%, *n* = 27,311). Age distribution analysis revealed the largest proportion of cases occurred among middle‐aged adults (45–64 years: *n* = 14,339; 30.43%), followed by young adults (25–44 years: *n* = 13,042; 27.68%). Younger age groups accounted for progressively smaller proportions (15–24 years: 21.06%; 5–14 years: 2.52%; < 5 years: 0.44%), with elderly patients (≥ 65 years) representing 17.88% of cases. The overall mean age was 43.94 years (SD = 20.08). Propensity score matching successfully balanced baseline characteristics between RTI cases and controls, with no significant differences observed in sex distribution, injury patterns, hospitalization outcomes, age stratification, comorbidity profiles (including catastrophic illness, hypertension, COPD, renal disease, and epilepsy), or residential urbanization levels (Table 1). As detailed in Supplementary Table S2, the median follow‐up time for the entire cohort was 6.91 years, with no significant difference between groups (*p* = 0.346). Despite similar follow‐up, the median time to a mental health diagnosis was significantly shorter for RTI inpatients (5.83 years) compared to controls (6.14 years, *p* < 0.001).

**TABLE 1:** Sex differences in epidemiologic characteristics and prognosis outcomes among RTI inpatients.

	Sex					Prognosis				
	Male (*n* = 27,311)		Female (*n* = 19,814)			Survive (*n* = 46,415)		Death (*n* = 710)		
	*n*	%	*n*	%	*p*	*n*	%	*n*	%	*p*
RTI inpatient					< 0.001					0.833
Driver of motor vehicle	22,040	80.70	14,861	75.00		36,359	78.33	542	76.34	
Passenger in motor vehicle	1366	5.00	396	2.00		1735	3.74	27	3.80	
Motorcyclist	792	2.90	1,487	7.50		2244	4.83	35	4.93	
Passenger on motorcycle	410	1.50	515	2.60		910	1.96	15	2.11	
Pedal cyclist	1202	4.40	853	4.31		2020	4.35	35	4.93	
Pedestrian	1255	4.60	1504	7.59		2,709	5.84	50	7.04	
Others	246	0.90	198	1.00		438	0.94	6	0.85	
Prognosis					< 0.001	N.A.	N/A	N.A.	N/A	N/A
Survive	26,819	98.20	19,596	98.90		N.A.	N/A	N.A.	N/A	N/A
Death	492	1.80	218	1.10		N.A.	N/A	N.A.	N/A	N/A
Sex	N.A.	N/A	N.A.	N/A	N/A					< 0.001
Male	N.A.	N/A	N.A.	N/A	N/A	26,887	57.93	424	59.72	
Female	N.A.	N/A	N.A.	N/A	N/A	19,528	42.07	286	40.28	
Age groups (years)					< 0.001					0.985
< 5	109	0.40	99	0.50		205	0.44	3	0.42	
5–14	710	2.60	476	2.40		1168	2.52	18	2.54	
15–24	6418	23.50	3507	17.70		9773	21.06	152	21.41	
25–44	8029	29.40	5013	25.30		12,838	27.66	204	28.73	
45–64	7047	25.80	7292	36.80		14,128	30.44	211	29.72	
≧ 65	4998	18.30	3427	17.30		8303	17.89	122	17.18	
Low‐income household	464	1.70	337	1.70	0.988	784	1.69	17	2.39	0.143
Catastrophic illness	1174	4.30	535	2.70	< 0.001	1663	3.58	46	6.48	< 0.001
Comorbidities										
DM	4971	18.20	3527	17.80	0.264	8280	17.84	218	30.70	< 0.001
HTN	4861	17.80	3309	16.70	0.002	7,986	17.21	184	25.92	< 0.001
Hyperlipidemia	4151	15.20	2,952	14.90	0.368	6,946	14.96	157	22.11	< 0.001
MI	2240	8.20	1585	8.00	0.427	3696	7.96	129	18.17	< 0.001
CVD	2950	10.80	2,080	10.50	0.292	4924	10.61	106	14.93	< 0.001
COPD	3387	12.40	2318	11.70	0.021	5590	12.04	115	16.20	0.001
Pneumonia	1420	5.20	951	4.80	0.052	2,305	4.97	66	9.30	< 0.001
Respiratory failure	355	1.30	218	1.10	0.055	544	1.17	29	4.08	< 0.001
CKD	2622	9.60	1605	8.10	< 0.001	4,141	8.92	86	12.11	0.004
Epilepsy	273	1.00	159	0.80	0.028	421	0.91	11	1.55	0.106
Season					0.851					0.582
Spring	6582	24.10	4795	24.20		11,216	24.16	161	22.68	
Summer	6937	25.40	4991	25.19		11,756	25.33	172	24.23	
Autumn	6633	24.29	4870	24.58		11,323	24.40	180	25.35	
Winter	7159	26.21	5158	26.03		12,120	26.11	197	27.75	
Urbanization level					< 0.001					0.018
1 (The highest)	7034	25.76	5856	29.55		12,666	27.29	224	31.55	
2	10,345	37.88	7857	39.65		17,929	38.63	273	38.45	
3	9932	36.37	6,101	30.79		15,820	34.08	213	30.00	
Level of care					0.625					0.004
Hospital center	8248	30.20	5964	30.10		13,959	30.07	253	35.63	
Regional hospital	9750	35.70	7011	35.38		16,518	35.59	243	34.23	
Local hospital	9313	34.10	6839	34.52		15,938	34.34	214	30.14	
	Sex					Prognosis				
	Male (*n* = 27,311)		Female (*n* = 19,814)			Survive (*n* = 46,415)		Death (*n* = 710)		
	Mean	SD	Mean	SD	*p*	Mean	SD	Mean	SD	*p*
Age (years)	42.81	19.96	45.49	20.14	< 0.001	43.97	20.10	42.16	18.35	0.017

*Note*: *p*: Chi‐square / Fisher exact test (categorical variable) and *t*‐test (continuous variable).

**Abbreviation**: N/A = not applicable.

### 3.2 Kaplan–Meier Model for the Cumulative Risk of Mental Health Issues

**FIGURE 2:**
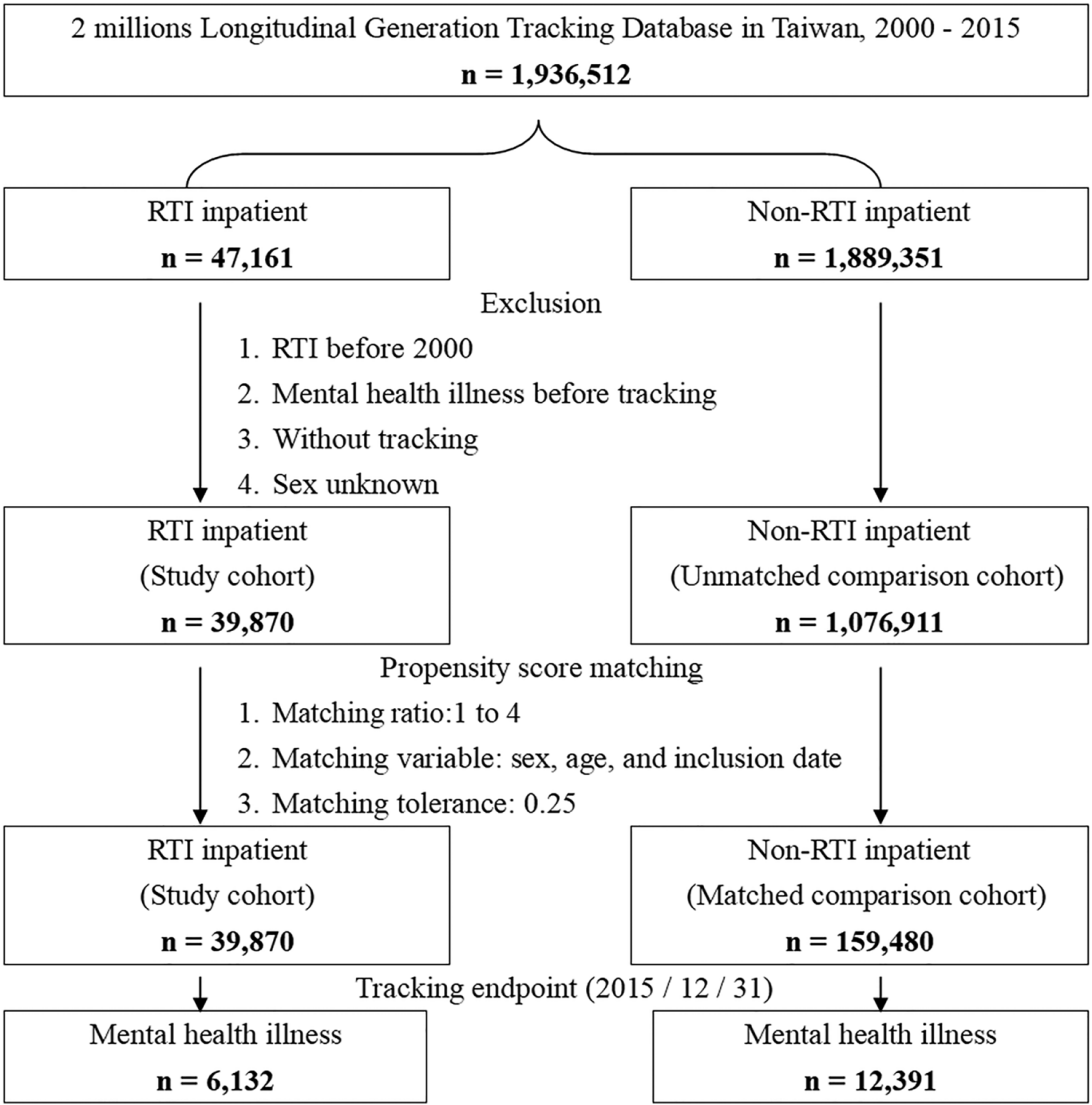
Participant selection and propensity score matching flowchart.

Kaplan–Meier survival analysis demonstrated significantly different cumulative incidence curves for mental health disorders between RTI patients and matched controls throughout the 15‐year follow‐up period (log‐rank test: *p* < 0.001). The divergence in mental health risk trajectories was evident early in follow‐up and persisted over time (Table 2, Figure 2). Moreover, the year‐by‐year cumulative number of events for both groups is detailed in Supplementary Table S3.

**TABLE 2:** Factors of RTI inpatient mortality by using multivariable conditional logistic regression.

	Crude OR	95% CI	95% CI	*p*	aOR	95% CI	95% CI	*p*
RTI inpatient								
Driver of motor vehicle	Reference				Reference			
Passenger in motor vehicle	1.043	1.038	1.049	0.024	1.028	1.011	1.045	0.044
Motorcyclist	1.046	1.041	1.051	0.025	1.031	1.014	1.047	0.043
Passenger on motorcycle	1.104	1.096	1.112	< 0.001	1.088	1.068	1.108	0.016
Pedal cyclist	1.160	1.154	1.165	< 0.001	1.143	1.124	1.161	< 0.001
Pedestrian	1.234	1.229	1.239	< 0.001	1.216	1.197	1.234	< 0.001
Others	0.920	0.909	0.931	0.004	0.906	0.885	0.928	0.014
Gender								
Male	1.076	1.074	1.077	0.012	1.060	1.046	1.073	0.027
Female	Reference				Reference			
Age groups (years)								
< 5	Reference				Reference			
5–14	1.052	1.045	1.059	0.020	1.036	1.018	1.055	0.040
15–24	1.062	1.059	1.064	0.017	1.046	1.032	1.060	0.034
25–44	1.084	1.082	1.087	0.006	1.068	1.054	1.083	0.023
45–64	1.020	1.018	1.022	0.039	1.005	0.992	1.018	0.058
≧ 65	1.004	1.001	1.007	0.044	0.989	0.975	1.003	0.072
Low‐income household ^@^	1.419	1.409	1.429	< 0.001	1.398	1.373	1.424	< 0.001
Catastrophic illness ^@^	1.841	1.833	1.849	< 0.001	1.814	1.786	1.842	< 0.001
Comorbidities ^@^								
DM	2.014	2.011	2.017	< 0.001	1.984	1.959	2.010	< 0.001
HTN	1.668	1.665	1.671	< 0.001	1.643	1.622	1.665	< 0.001
Hyperlipidemia	1.600	1.596	1.603	< 0.001	1.576	1.555	1.597	< 0.001
MI	2.513	2.508	2.519	< 0.001	2.476	2.443	2.510	< 0.001
CVD	1.469	1.465	1.473	< 0.001	1.447	1.427	1.468	< 0.001
COPD	1.403	1.400	1.407	< 0.001	1.382	1.364	1.402	< 0.001
Pneumonia	1.934	1.928	1.941	< 0.001	1.905	1.878	1.934	< 0.001
Respiratory failure	3.460	3.442	3.478	< 0.001	3.409	3.353	3.465	< 0.001
CKD	1.399	1.394	1.403	< 0.001	1.378	1.358	1.398	< 0.001
Epilepsy	1.701	1.686	1.716	< 0.001	1.676	1.642	1.710	< 0.001
Season								
Spring	Reference				Reference			
Summer	1.019	1.017	1.021	0.040	1.004	0.991	1.017	0.056
Autumn	1.106	1.103	1.108	< 0.001	1.090	1.074	1.104	0.013
Winter	1.130	1.128	1.132	< 0.001	1.113	1.099	1.128	< 0.001
Urbanization level								
1 (The highest)	1.308	1.306	1.310	< 0.001	1.289	1.272	1.305	< 0.001
2	1.129	1.127	1.131	< 0.001	1.112	1.098	1.127	0.001
3	Reference				Reference			
Level of care								
Hospital center	1.344	1.341	1.346	< 0.001	1.324	1.306	1.341	< 0.001
Regional hospital	1.094	1.092	1.096	0.003	1.078	1.064	1.092	0.018
Local hospital	Reference				Reference			

^a^ Reference: Without.

Abbreviations: Adjusted OR = Adjusted variables listed in the table. CI = confidence interval.

### 3.3 Comparisons of the Prevalence of Mental Health Issues

**FIGURE 3:**
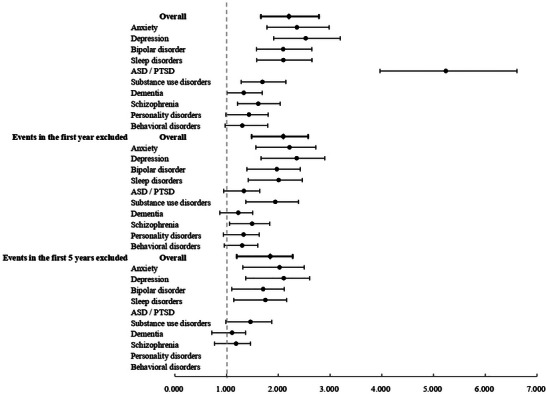
Forest plot of sensitivity analyses for mental health outcomes.

The longitudinal analysis revealed a substantially higher burden of mental health conditions among RTI survivors compared to controls. At the end of follow‐up, 13.01% (*n* = 6,132/47,161) of the RTI cohort had received mental health diagnoses, versus only 1.15% (*n* = 12,391/1,076,911) in the control population (*p* < 0.001). This elevated risk persisted consistently across all demographic and clinical strata, including sex, age groups, income levels, seasonal periods, comorbidity status, residential urbanization levels, and hospital accreditation categories (all subgroup *p*‐values <0.001; Table 3; Figure 3). Detailed data are presented in Supplementary Table S4.

**TABLE 3:** Characteristics of study in the baseline and endpoint.

	Baseline					Endpoint				
RTI inpatient	With (*n* = 39,870)		Without (*n* = 159,480)			With (*n* = 39,870)		Without (*n* = 159,480)		
	*n*	%	*n*	%	*p*	*n*	%	*n*	%	*p*
Mental health illness	N/A	N/A	N/A	N/A	N/A	6132	15.38	12,391	7.77	< 0.001
Sex					0.999					0.999
Male	23,106	57.95	92,424	57.95		23,106	57.95	92,424	57.95	
Female	16,764	42.05	67,056	42.05		16,764	42.05	67,056	42.05	
Age groups (years)					0.999					< 0.001
< 5	176	0.44	704	0.44		164	0.41	683	0.43	
5–14	1003	2.52	4,012	2.52		998	2.50	4001	2.51	
15–24	8397	21.06	33,588	21.06		8372	21.00	33,517	21.02	
25–44	11,034	27.67	44,136	27.67		11,025	27.65	40,126	25.16	
45–64	12,131	30.43	48,524	30.43		12,121	30.40	48,302	30.29	
≧ 65	7129	17.88	28,516	17.88		7190	18.03	32,851	20.60	
Low‐income household	678	1.70	2,864	1.80	0.204	684	1.72	2871	1.80	0.262
Catastrophic illness	1445	3.62	5,102	3.20	< 0.001	1456	3.65	5,122	3.21	< 0.001
Comorbidities										
DM	7188	18.03	28,111	17.63	0.060	7206	18.07	22,512	14.12	< 0.001
HTN	6912	17.34	26,350	16.52	< 0.001	6974	17.49	26,378	16.54	< 0.001
Hyperlipidemia	6009	15.07	23,195	14.54	0.008	6012	15.08	23,202	14.55	0.002
MI	3236	8.12	12,702	7.96	0.318	3,245	8.14	12,771	8.01	0.389
CVD	4256	10.67	16,863	10.57	0.558	4298	10.78	16,878	10.58	0.254
COPD	4827	12.11	18,667	11.70	0.026	4903	12.30	18,704	11.73	0.002
Pneumonia	2003	5.02	7251	4.55	< 0.001	2,015	5.05	7269	4.56	< 0.001
Respiratory failure	485	1.22	1925	1.21	0.878	497	1.25	1930	1.21	0.557
CKD	3576	8.97	13,306	8.34	< 0.001	3588	9.00	13,324	8.35	< 0.001
Epilepsy	361	0.91	1420	0.89	0.766	365	0.92	1422	0.89	0.656
Season					0.999					0.009
Spring	9626	24.14	38,504	24.14		9634	24.16	37,582	23.57	
Summer	10,090	25.31	40,360	25.31		10,025	25.14	41,256	25.87	
Autumn	9731	24.41	38,924	24.41		9786	24.54	38,971	24.44	
Winter	10,423	26.14	41,692	26.14		10,425	26.15	41,671	26.13	
Urbanization level					0.015					0.039
1 (The highest)	10,906	27.35	42,618	26.71		11,012	27.62	43,687	27.39	
2	15,402	38.63	62,675	39.28		14,896	37.36	60,678	38.05	
3	13,562	34.02	54,248	34.00		13,962	35.02	55,115	34.56	
Level of care					< 0.001					< 0.001
Hospital center	12,024	30.16	50,025	31.37		12,135	30.44	49,983	31.34	
Regional hospital	14,181	35.57	57,711	36.19		14,106	35.38	56,371	35.35	
Local hospital	13,665	34.27	51,744	32.45		13,629	34.18	53,126	33.31	
	Baseline					Endpoint				
RTI inpatient	With (*n* = 39,870)		Without (*n* = 159,480)			With (*n* = 39,870)		Without (*n* = 159,480)		
	Mean	SD	Mean	SD	*p*	Mean	SD	Mean	SD	*p*
Age (years)	41.15	15.29	41.16	15.32	0.907	46.85	20.27	47.91	21.19	< 0.001

*Note*: *p*: Chi‐square / Fisher exact test (categorical variable) and *t*‐test (continuous variable).

**Abbreviation**: N/A = Not applicable

### 3.4 Hazard Ratios Analysis of Developing Mental Health Illness After Hospitalization due to RTI

In the Cox proportional hazards regression analysis, after adjusting for sex, age, monthly income, season, comorbidities, urbanization level of residence, and hospital accreditation level, patients hospitalized due to RTIs had a significantly higher risk of developing mental health conditions compared to controls, with an adjusted hazard ratio (HR) of 2.204 (95% CI: 1.663–2.785, p < 0.001).

We further analyzed the risk according to the type of road user involved in the crash (Supplementary Table S5). Compared to the control group, all specific types of road users—including drivers of motor vehicles, passengers, motorcyclists, pedal cyclists, and pedestrians—exhibited a significantly elevated risk of mental health disorders (aHR ranging from 2.18 to 2.45, all *p* < .001). Among these, pedestrians were at the highest risk (aHR = 2.45, 95% CI: 1.84–3.01). The risk for the group categorized as “Other” was also significantly increased, albeit to a lesser magnitude.

**FIGURE 4:**
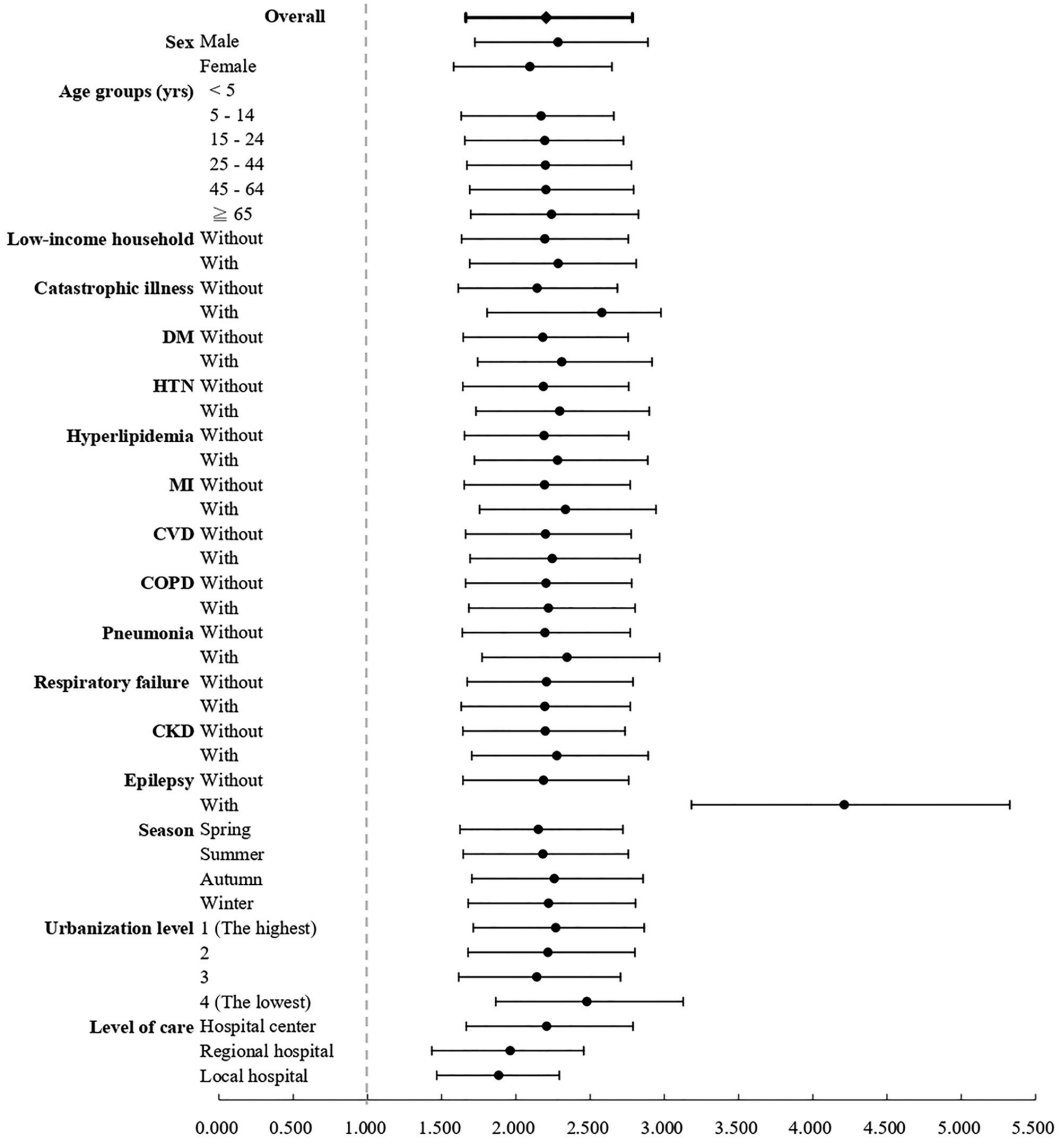
Subgroup analysis of the association between RTI and mental health outcomes.

When mental health conditions were analyzed by type, patients in the RTI group exhibited significantly elevated risks for anxiety, depression, bipolar disorder, sleep disorders, acute stress disorder/post‐traumatic stress disorder (PTSD), substance use disorders, dementia, schizophrenia, personality disorders, and behavioral disorders compared to the control group (Table 4; Figure 4).

**TABLE 4:** Factors of mental health illness by using Cox regression.

	Crude HR	95% CI	95% CI	*p*	aHR	95% CI	95% CI	*p*
RTI inpatient ^@^	2.777	1.987	3.816	< 0.001	2.204	1.663	2.785	< 0.001
Gender								
Male	1.845	1.172	2.701	< 0.001	1.465	1.024	1.972	0.038
Female	Reference				Reference			
Age groups (years)								
< 5	0.000	—	—	0.999	0.000	—	—	0.999
5–14	Reference				Reference			
15–24	1.621	1.099	1.967	0.001	1.297	1.020	1.435	0.040
25–44	1.648	1.297	2.195	< 0.001	1.303	1.124	1.597	< 0.001
45–64	1.970	1.384	2.444	< 0.001	1.567	1.205	1.787	< 0.001
≧ 65	2.293	1.542	2.876	< 0.001	1.825	1.345	2.101	< 0.001
Low‐income household ^@^	1.637	1.188	2.162	< 0.001	1.301	1.036	1.579	0.032
Catastrophic illness ^@^	3.456	2.287	4.452	< 0.001	2.650	1.989	3.256	
Comorbidities ^@^								
DM	2.106	1.495	2.578	< 0.001	1.672	1.303	1.882	< 0.001
HTN	1.995	1.452	2.463	< 0.001	1.586	1.264	1.789	< 0.001
Hyperlipidemia	2.274	1.610	2.731	< 0.001	1.808	1.403	1.997	< 0.001
MI	1.501	1.001	2.059	0.049	1.203	0.876	1.503	0.128
CVD	1.956	1.183	2.712	< 0.001	1.555	1.035	1.978	0.033
COPD	2.103	1.412	2.730	< 0.001	1.672	1.235	1.989	< 0.001
Pneumonia	1.706	1.201	2.175	< 0.001	1.356	1.060	1.588	0.020
Respiratory failure	1.124	0.725	1.860	0.277	0.896	0.571	1.265	0.426
CKD	2.672	1.894	3.101	< 0.001	2.506	1.865	2.970	< 0.001
Epilepsy	4.297	2.584	5.556	< 0.001	3.359	2.265	4.054	< 0.001
Season								
Spring	Reference				Reference			
Summer	1.393	0.789	2.437	0.207	1.106	0.686	1.780	0.324
Autumn	2.462	1.578	3.397	< 0.001	1.933	1.372	2.476	< 0.001
Winter	2.125	1.189	3.104	< 0.001	1.678	1.034	2.265	0.033
Urbanization level								
1 (The highest)	2.450	1.789	2.904	< 0.001	1.835	1.405	2.056	< 0.001
2	1.825	1.457	2.291	< 0.001	1.462	1.265	1.673	< 0.001
3	Reference				Reference			
Level of care								
Hospital center	3.326	2.678	4.083	< 0.001	2.661	2.320	2.974	< 0.001
Regional hospital	2.876	2.230	3.462	< 0.001	2.350	1.878	2.526	< 0.001
Local hospital	Reference				Reference			

^@^ Reference: Without.

**Abbreviations**: Adjusted OR = Adjusted variables listed in the table. CI = confidence interval.

## 4 Discussion

### 4.1 Association Between RTIs Caused Hospitalization and the Risk of Mental Health Illness

This study found that patients hospitalized for RTIs faced a significantly higher risk of developing mental health disorders compared to matched controls. After adjusting for demographic, socioeconomic, and clinical confounders, the risk was more than doubled (adjusted HR = 2.20, 95% CI: 1.66–2.79, *p* < 0.001). The Kaplan–Meier analysis confirmed this association, with a clear divergence in cumulative incidence over the 15‐year follow‐up (log‐rank *p* < 0.001).

**FIGURE 5:**
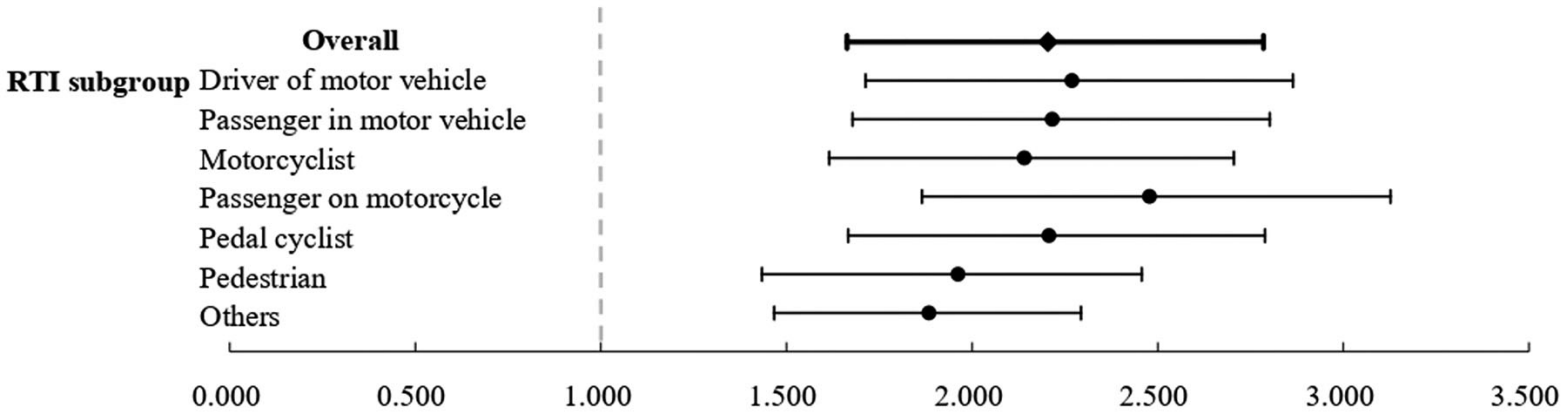
Subgroup analysis by type of road traffic injury.

The increased risk spanned multiple psychiatric conditions, including PTSD, depression, anxiety, substance use disorders, and psychotic disorders. These findings highlight the need for early mental health screening and long‐term monitoring in RTI survivors to address both immediate and delayed psychological consequences (Figure 5).

### 4.2 Association Between Relationship of RTIs Caused Hospitalization and the Risk of Mental Health Illness

Previous studies have identified depression, anxiety disorders (including phobias), and post‐traumatic stress symptoms as common psychological sequelae following traffic‐related injuries (Yu et al. 2025; Bryant et al. 2010; Grant et al. 2008; Kenardy et al. 2018). Reported prevalence rates of mental health disorders post‐injury vary widely, with some research indicating rates between 30% and 50% (Bryant et al. 2010; Guest et al. 2018; Kenardy et al. 2018; Mayou and Bryant 2001). Without timely treatment, these symptoms may evolve into more serious psychiatric conditions such as major depressive disorder (MDD), PTSD, panic disorder, and generalized anxiety disorder (Grant et al. 2008; Kenardy et al. 2018). Moreover, affected individuals are at increased risk of developing behavioral issues, including aggression and substance abuse (Beck and Coffey 2007; deRoon‐Cassini et al. 2010). Notably, motor vehicle crashes have been linked with current major depressive episodes and alcohol misuse, particularly among younger and older adolescent populations (Williams et al. 2015). In line with these findings, a recent review highlighted that psychological consequences after road traffic injuries can persist long term, with heterogeneous recovery trajectories and significant risks of anxiety, depression, and PTSD. Additionally, some studies emphasize that the overall cost of care—including medical treatment and rehabilitation—may nearly double when psychological distress is factored in (Chan et al. 2003).

These findings highlight the importance of early mental health screening and intervention in individuals recovering from traffic injuries.

### 4.3 Possible Mechanisms for the Increased Risk of Mental Health Illness Injuries in Adults With Hospitalization due to RTI

The increased risk of mental health disorders observed among adults hospitalized for RTIs likely arises from a complex interplay of multiple pathogenic mechanisms. The immediate psychological impact of traumatic collisions may overwhelm normal stress response systems, creating a vulnerable state for developing persistent psychiatric conditions. Many patients experience profound distress during the accident itself, with intrusive memories and heightened arousal potentially laying the foundation for PTSD. The subsequent physical recovery period often introduces additional risk factors, including chronic pain, restricted mobility, and prolonged dependence on analgesics, all of which are independently associated with mood disturbances. Furthermore, the disruption to daily life during hospitalization and rehabilitation can significantly alter social dynamics and occupational functioning, potentially leading to financial strain, role changes within families, and reduced social support—factors known to exacerbate psychological distress. At the biological level, the substantial physiological stress of severe injury may dysregulate neuroendocrine systems, particularly the hypothalamic‐pituitary‐adrenal axis, creating lasting alterations in stress response and emotional regulation. This combination of acute trauma, ongoing physical challenges, social consequences, and biological changes appears to create a perfect storm for the development of diverse mental health conditions following serious motor vehicle accidents.

### 4.4 Limitation

Several limitations of this study should be noted. First, the dataset did not include information on participants’ previous psychiatric history or family history of mental illness, both of which are significant risk factors that could potentially confound the observed relationships. Second, although the use of a national health insurance database enabled a large sample size with broad coverage, the data were limited to administrative and diagnostic codes. As a result, detailed contextual factors surrounding the RTIs—such as involvement of alcohol or drug use, driver fatigue, or other risky behaviors—could not be assessed. Third, the findings may not be generalizable beyond the Taiwanese population and healthcare system, as cultural attitudes, healthcare access, and mental illness prevalence vary widely between countries. In particular, baseline rates and detection of psychiatric disorders differ internationally, limiting the applicability of these results elsewhere. Finally, the relatively short follow‐up period may have hindered identification of delayed‐onset psychiatric conditions, including chronic PTSD or major depressive disorder, which can manifest months or even years post‐injury. Thus, further longitudinal research is necessary to fully understand the long‐term mental health impact of motor vehicle trauma.

### 4.5 Implications for Trauma Care as Mental Health Screening and Early Intervention

The present findings suggest that hospitalization for RTIs substantially increases the risk of subsequent mental health disorders. Incorporating standardized mental health screening at the time of admission and during follow‐up could enable early identification of patients at risk for mental health illness. Trauma services may also benefit from embedding multidisciplinary teams, including psychiatrists, psychologists, and social workers, to provide timely psychological support alongside physical rehabilitation. Early intervention—such as psychoeducation, brief cognitive‐behavioral therapy, or referral—may mitigate long‐term psychiatric morbidity and reduce the overall burden on healthcare systems. From a policy perspective, developing structured care pathways that mandate psychological assessment after severe RTIs could facilitate more holistic recovery and decrease healthcare expenditures associated with untreated psychiatric sequelae. Future research should evaluate the cost‐effectiveness and feasibility of such integrated models in different healthcare settings.

## 5 Conclusion

This study provides robust evidence linking hospitalization for road traffic injuries (RTIs) to an increased long‐term risk of developing a wide spectrum of mental health disorders. Across a large, nationwide cohort and a 15‐year follow‐up, RTI survivors consistently demonstrated significantly higher rates of psychiatric diagnoses compared to matched controls, with the divergence in risk evident early and persisting throughout the observation period. Adjusted analyses further confirmed that hospitalization due to RTIs was independently associated with a more than twofold higher hazard of subsequent mental illness, spanning common conditions such as anxiety and depression, as well as severe disorders including schizophrenia, bipolar disorder, and dementia. These findings highlight the enduring psychological burden that follows severe traffic‐related injuries and underscore the importance of incorporating systematic mental health assessments and targeted interventions into post‐injury care and rehabilitation programs. Future longitudinal and prospective studies are warranted to clarify underlying causal pathways, identify vulnerable subgroups, and evaluate preventative and therapeutic strategies aimed at mitigating the long‐term psychiatric sequelae of RTIs.

## Author Contributions

Conceptualization: Yu‐An Chen, Wu‐Chien Chien, and Chi‐Hsiang Chung. Methodology: Yu‐An Chen, Wu‐Chien Chien, and Chi‐Hsiang Chung. Software: Chi‐Hsiang Chung. Data curation: Wu‐Chien Chien and Chi‐Hsiang Chung. Investigation: Wu‐Chien Chien, Li‑Yun Fann, Ying‐Che Huang, Tsu‐Hsuan Weng, and Chun‐Teng Tsai. Validation: Wu‐Chien Chien, Li‑Yun Fann, Ying‐Che Huang, Tsu‐Hsuan Weng, and Chun‐Teng Tsai. Formal analysis: Chi‐Hsiang Chung. Supervision: Wu‐Chien Chien, Li‑Yun Fann, Ying‐Che Huang, Chi‐Hsiang Chung, Tsu‐Hsuan Weng, Chun‐Teng Tsai. Funding acquisition: Wu‐Chien Chien. Visualization: Chi‐Hsiang Chung. Project administration: Wu‐Chien Chien, Li‑Yun Fann, Ying‐Che Huang, Chi‐Hsiang Chung, Tsu‐Hsuan Weng, Chun‐Teng Tsai. Resources: Wu‐Chien Chien, Chi‐Hsiang Chung. Writing—original draft: Yu‐An Chen. Writing—review & editing: Yu‐An Chen.

## Funding

This study was supported by the Tri‐Service General Hospital Research Foundation(TSGH‐B‐114022; TSGH‐A‐114010; TSGH‐D‐114196), and the sponsor has no role in a study design, data collection and analysis, decision to publish, or preparation of the manuscript. The study was approved by the Ethucs Review Board of Tri‐Service General Hospital, National defense Medical Center(TSGHIRB, No. E202516024)

## Conflicts of Interest

The authors declare no conflicts of interest.

## Supporting information




**Table S1** ICD‐9‐CM and definition

**Table S2** Years of follow‐up and time to events

**Table S3** Kaplan–Meier for cumulative mental health illness stratified by RTI inpatient with log‐rank test

**Table S4** Factors of mental health illness stratified by variables listed in the table by using Cox regression and Bonferroni correction for multiple comparisons

**Table S5** Factors of mental health illness among different RTI inpatients by using Cox regression and Bonferroni correction for multiple comparisons

**Table S6** Sensitivity analysis for factors of mental health illness subgroups by using Cox regression and Bonferroni correction for multiple comparisons

## Data Availability

The data used in this study were obtained from the NHIRD, which is maintained by the Health and Welfare Data Science Center (HWDC), Ministry of Health and Welfare, Taiwan. Due to legal restrictions and the personal data protection regulations of Taiwan, the dataset is not publicly available. Qualified researchers can apply for access to the NHIRD through the HWDC (https://dep.mohw.gov.tw/dos/np‐2497‐113.html).
