# Supplementary material for: The Risk of Road Traffic Injuries Caused Hospitalization and the Risk of Mental Health Illness: A Nationwide, Matched‐Cohort, Population‐Based Study in Taiwan
Source: Brain Behav. 2025 Nov 10;15(11):e70993. doi: 10.1002/brb3.70993 (PMC12602460; doi:10.1002/brb3.70993)
Supplement: Supplementary file 1 — Table S1 ICD‐9‐CM and definition [file BRB3-15-e70993-s005.docx]

**Table S1.** ICD-9-CM and definition

|  | ICD-9-CM and definition |
| --- | --- |
| Study population: RTI inpatient | Hospitalizations with any of the following as a primary or secondary diagnosis; in cases of multiple records, the one with the earliest diagnosis ranking was selected |
| Driver of motor vehicle | E810.x0 - E819.x0 |
| Passenger in motor vehicle | E810.x1 - E819.x1 |
| Motorcyclist | E810.x2 - E819.x2 |
| Passenger on motorcycle | E810.x3 - E819.x3 |
| Pedal cyclist | E810.x6 - E819.x6 |
| Pedestrian | E810.x7 - E819.x7 |
| Others | E810 - E819, The aforementioned were excluded |
| Event: Mental health illness | Having any of the following as a primary or secondary diagnosis in three or more psychiatric or neurological outpatient or emergency department visits, or in any hospitalization; in cases with multiple records, the diagnosis with the highest priority (earliest in order) was selected |
| Anxiety | 300 |
| Depression | 296.2 - 296.3, 300.4, 311 |
| Bipolar disorder | 296.0, 296.4 - 296.8 |
| Sleep disorders | 307.4, 780.5 |
| ASD/PTSD | 308, 309.81 |
| Substance use disorders | 291, 292, 303.3, 303.9, 304, 305 |
| Dementia | 290.0 - 290.4, 290.8, 290.9, 331.0 |
| Schizophrenia | 295 |
| Personality disorders | 301 |
| Behavioral disorders | 312 |
| Comorbidities | Primary and secondary diagnoses, in 3 outpatient or emergency visits, or during hospitalization; the study start period is from before enrollment to the start of the study, and the study end period is from before enrollment to the end of the study. |
| DM | 250 |
| HTN | 401 - 405 |
| Hyperlipidemia | 272 |
| MI | 410 - 412 |
| CVD | 480 - 489 |
| COPD | 490 - 496 |
| Pneumonia | 480 - 488, 507 |
| Respiratory failure | 518.8 |
| CKD | 580 - 589 |
| Epilepsy | 345, 780.3 |
