# Supplementary material for: The Risk of Road Traffic Injuries Caused Hospitalization and the Risk of Mental Health Illness: A Nationwide, Matched‐Cohort, Population‐Based Study in Taiwan
Source: Brain Behav. 2025 Nov 10;15(11):e70993. doi: 10.1002/brb3.70993 (PMC12602460; doi:10.1002/brb3.70993)
Supplement: Supplementary file 2 — Table S2 Years of follow‐up and time to events [file BRB3-15-e70993-s006.docx]

**Table S2.** Years of follow-up and time to events

|  | RTI inpatient | Minimum | Median | Maximum | Mean **±** SD | *p* |
| --- | --- | --- | --- | --- | --- | --- |
| Tracking years | Overall (n = 199,350) | 0.06 | 6.91 | 15.89 | 7.85 ± 5.69 | 0.346 |
|  | With (n = 39,870) | 0.06 | 6.89 | 15.84 | 7.83 ± 5.65 |  |
|  | Without (n = 159,480) | 0.06 | 6.94 | 15.89 | 7.86 ± 5.70 |  |
| Years to mental health illness | Overall (n = 199,350) | 0.12 | 5.98 | 15.87 | 6.15 ± 4.92 | < 0.001 |
|  | With (n = 39,870) | 0.12 | 5.83 | 15.80 | 5.91 ± 4.72 |  |
|  | Without (n = 159,480) | 0.12 | 6.14 | 15.87 | 6.21 ± 4.97 |  |

*p*: t-test
