# Supplementary material for: The Risk of Road Traffic Injuries Caused Hospitalization and the Risk of Mental Health Illness: A Nationwide, Matched‐Cohort, Population‐Based Study in Taiwan
Source: Brain Behav. 2025 Nov 10;15(11):e70993. doi: 10.1002/brb3.70993 (PMC12602460; doi:10.1002/brb3.70993)
Supplement: Supplementary file 3 — Table S3 Kaplan–Meier for cumulative mental health illness stratified by RTI inpatient with log‐rank test [file BRB3-15-e70993-s004.docx]

**Table S3.** Kaplan-Meier for cumulative mental health illness stratified by RTI inpatient with log-rank test

| Tracking years | RTI inpatient  (n = 39,870) | Non-RTI inpatient  (n = 159,480) | *p* |
| --- | --- | --- | --- |
| 1 | 513 | 880 | < 0.001 |
| 2 | 1,024 | 1,724 | < 0.001 |
| 3 | 1,539 | 2,621 | < 0.001 |
| 4 | 2,027 | 3,405 | < 0.001 |
| 5 | 2,642 | 4,316 | < 0.001 |
| 6 | 3,068 | 5,207 | < 0.001 |
| 7 | 3,409 | 6,196 | < 0.001 |
| 8 | 3,741 | 6,972 | < 0.001 |
| 9 | 4,083 | 7,748 | < 0.001 |
| 10 | 4,420 | 8,522 | < 0.001 |
| 11 | 4,766 | 9,297 | < 0.001 |
| 12 | 5,105 | 10,079 | < 0.001 |
| 13 | 5,449 | 10,851 | < 0.001 |
| 14 | 5,780 | 11,626 | < 0.001 |
| 15 | 6,132 | 12,391 | < 0.001 |

*p*: Log-rank test
