# Supplementary material for: The Risk of Road Traffic Injuries Caused Hospitalization and the Risk of Mental Health Illness: A Nationwide, Matched‐Cohort, Population‐Based Study in Taiwan
Source: Brain Behav. 2025 Nov 10;15(11):e70993. doi: 10.1002/brb3.70993 (PMC12602460; doi:10.1002/brb3.70993)
Supplement: Supplementary file 4 — Table S4 Factors of mental health illness stratified by variables listed in the table by using Cox regression and Bonferroni correction for multiple comparisons [file BRB3-15-e70993-s001.doc]

**Table S4.** Factors of mental health illness stratified by variables listed in the table by using Cox regression and Bonferroni correction for multiple comparisons

|  | RTI inpatient | With (n = 39,870) | | | Without (n = 159,480) | | | With vs. Without (Reference) | | | |
| --- | --- | --- | --- | --- | --- | --- | --- | --- | --- | --- | --- |
|  |  | Events | PYs | Rate | Events | PYs | Rate | aHR | 95%CI | 95%CI | *p* |
|  | Overall | 6,132 | 313,309.27 | 1,957.17 | 12,391 | 1,253,296.73 | 988.67 | 2.204 | 1.663 | 2.785 | < 0.001 |
| Sex | Male | 3,672 | 180,576.35 | 2,033.49 | 7,201 | 726,375.11 | 991.36 | 2.284 | 1.724 | 2.889 | < 0.001 |
|  | Female | 2,460 | 132,732.92 | 1,853.35 | 5,190 | 526,921.62 | 984.97 | 2.095 | 1.581 | 2.647 | < 0.001 |
| Age groups (yrs) | < 5 | 0 | 1,288.75 | 0.00 | 0 | 5,367.92 | 0.00 | - | - | - | - |
|  | 5 - 14 | 151 | 7,874.26 | 1,917.64 | 308 | 31,442.47 | 979.57 | 2.170 | 1.632 | 2.659 | < 0.001 |
|  | 15 - 24 | 1,283 | 65,789.44 | 1,950.16 | 2,605 | 263,297.17 | 989.38 | 2.195 | 1.656 | 2.724 | < 0.001 |
|  | 25 - 44 | 1,695 | 86,637.25 | 1,956.43 | 3,124 | 315,336.24 | 990.69 | 2.199 | 1.671 | 2.778 | < 0.001 |
|  | 45 - 64 | 1,872 | 95,250.21 | 1,965.35 | 3,774 | 379,588.30 | 994.24 | 2.202 | 1.689 | 2.793 | < 0.001 |
|  | ≧ 65 | 1,131 | 56,469.36 | 2,002.86 | 2,580 | 258,264.63 | 998.98 | 2.240 | 1.697 | 2.825 | < 0.001 |
| Low-income household | Without | 6,025 | 307,934.25 | 1,956.59 | 12,166 | 1,230,729.29 | 988.52 | 2.195 | 1.634 | 2.757 | < 0.001 |
|  | With | 107 | 5,375.02 | 1,990.69 | 225 | 22,567.44 | 997.01 | 2.285 | 1.689 | 2.811 | < 0.001 |
| Catastrophic illness | Without | 5,901 | 301,867.60 | 1,954.83 | 11,990 | 1,213,044.76 | 988.42 | 2.143 | 1.612 | 2.684 | < 0.001 |
|  | With | 231 | 11,441.67 | 2,018.94 | 401 | 40,251.97 | 996.22 | 2.579 | 1.806 | 2.977 | < 0.001 |
| DM | Without | 4,970 | 256,682.89 | 1,936.24 | 10,639 | 1,076,368.70 | 988.42 | 2.181 | 1.646 | 2.756 | < 0.001 |
|  | With | 1,162 | 56,626.38 | 2,052.05 | 1,752 | 176,928.03 | 990.23 | 2.309 | 1.743 | 2.917 | < 0.001 |
| HTN | Without | 5,007 | 258,506.06 | 1,936.90 | 10,324 | 1,045,998.37 | 987.00 | 2.185 | 1.642 | 2.760 | < 0.001 |
|  | With | 1,125 | 54,803.21 | 2,052.80 | 2,067 | 207,298.36 | 997.11 | 2.295 | 1.731 | 2.898 | < 0.001 |
| Hyperlipidemia | Without | 5,169 | 266,061.31 | 1,942.79 | 10,577 | 1,070,961.49 | 987.62 | 2.190 | 1.654 | 2.760 | < 0.001 |
|  | With | 963 | 47,247.96 | 2,038.18 | 1,814 | 182,335.24 | 994.87 | 2.281 | 1.721 | 2.888 | < 0.001 |
| MI | Without | 5,601 | 287,811.01 | 1,946.07 | 11,392 | 1,152,920.11 | 988.10 | 2.193 | 1.652 | 2.770 | < 0.001 |
|  | With | 531 | 25,498.26 | 2,082.50 | 999 | 100,376.62 | 995.25 | 2.334 | 1.756 | 2.943 | < 0.001 |
| CVD | Without | 5,454 | 279,537.70 | 1,951.08 | 11,067 | 1,120,598.71 | 987.60 | 2.200 | 1.661 | 2.777 | < 0.001 |
|  | With | 678 | 33,771.57 | 2,007.61 | 1,324 | 132,698.02 | 997.75 | 2.245 | 1.692 | 2.836 | < 0.001 |
| COPD | Without | 5,374 | 274,790.03 | 1,955.68 | 10,935 | 1,106,307.69 | 988.42 | 2.203 | 1.661 | 2.780 | < 0.001 |
|  | With | 758 | 38,519.24 | 1,967.85 | 1,456 | 146,989.04 | 990.55 | 2.219 | 1.684 | 2.803 | < 0.001 |
| Pneumonia | Without | 5,801 | 297,484.96 | 1,950.01 | 11,824 | 1,196,168.20 | 988.49 | 2.196 | 1.639 | 2.770 | < 0.001 |
|  | With | 331 | 15,824.31 | 2,091.72 | 567 | 57,128.53 | 992.50 | 2.345 | 1.772 | 2.968 | < 0.001 |
| Respiratory failure | Without | 6,055 | 309,411.63 | 1,956.94 | 12,239 | 1,238,120.53 | 988.51 | 2.206 | 1.672 | 2.789 | < 0.001 |
|  | With | 77 | 3,897.64 | 1,975.55 | 152 | 15,176.20 | 1,001.57 | 2.195 | 1.632 | 2.771 | < 0.001 |
| CKD | Without | 5,565 | 285,116.91 | 1,951.83 | 11,349 | 1,148,507.51 | 988.15 | 2.198 | 1.642 | 2.735 | < 0.001 |
|  | With | 567 | 28,192.36 | 2,011.18 | 1,042 | 104,789.22 | 994.38 | 2.276 | 1.703 | 2.891 | < 0.001 |
| Epilepsy | Without | 6,026 | 310,489.92 | 1,940.80 | 12,280 | 1,242,114.17 | 988.64 | 2.186 | 1.643 | 2.760 | < 0.001 |
|  | With | 106 | 2,819.35 | 3,759.73 | 111 | 11,182.56 | 992.62 | 4.212 | 3.183 | 5.327 | < 0.001 |
| Season | Spring | 1,412 | 75,706.95 | 1,865.09 | 2,842 | 295,343.22 | 962.27 | 2.151 | 1.623 | 2.721 | < 0.001 |
|  | Summer | 1,490 | 78,779.13 | 1,891.36 | 3,129 | 324,216.18 | 965.10 | 2.182 | 1.646 | 2.757 | < 0.001 |
|  | Autumn | 1,588 | 76,901.28 | 2,064.99 | 3,125 | 306,256.23 | 1,020.39 | 2.259 | 1.704 | 2.857 | < 0.001 |
|  | Winter | 1,642 | 81,921.91 | 2,004.35 | 3,295 | 327,481.10 | 1,006.16 | 2.220 | 1.679 | 2.806 | < 0.001 |
| Urbanization level | 1 (The highest) | 1,798 | 86,571.11 | 2,076.91 | 3,502 | 343,320.36 | 1,020.04 | 2.269 | 1.713 | 2.864 | < 0.001 |
|  | 2 | 2,291 | 117,056.82 | 1,957.17 | 4,722 | 479,846.17 | 984.07 | 2.216 | 1.678 | 2.802 | < 0.001 |
|  | 3 | 2,043 | 109,681.34 | 1,862.67 | 4,167 | 430,130.20 | 968.78 | 2.141 | 1.615 | 2.705 | < 0.001 |
| Level of care | Hospital center | 2,106 | 95,636.11 | 2,202.10 | 3,899 | 392,798.60 | 992.62 | 2.478 | 1.865 | 3.127 | < 0.001 |
|  | Regional hospital | 2,173 | 110,848.72 | 1,960.33 | 4,380 | 442,999.58 | 988.71 | 2.207 | 1.666 | 2.789 | < 0.001 |
|  | Local hospital | 1,853 | 106,824.44 | 1,734.62 | 4,112 | 417,498.55 | 984.91 | 1.962 | 1.434 | 2.457 | < 0.001 |

PYs = Person-years, Rate: per 100,000 PYs, aHR = Adjusted Hazard ratio: Adjusted for the variables listed in the table, CI = confidence interval
