# Supplementary material for: The Risk of Road Traffic Injuries Caused Hospitalization and the Risk of Mental Health Illness: A Nationwide, Matched‐Cohort, Population‐Based Study in Taiwan
Source: Brain Behav. 2025 Nov 10;15(11):e70993. doi: 10.1002/brb3.70993 (PMC12602460; doi:10.1002/brb3.70993)
Supplement: Supplementary file 5 — Table S5 Factors of mental health illness among different RTI inpatients by using Cox regression and Bonferroni correction for multiple comparisons [file BRB3-15-e70993-s003.doc]

**Table S5.** Factors of mental health illness among different RTI inpatient by using Cox regression and Bonferroni correction for multiple comparisons

| RTI inpatient | Populations | Events | PYs | Rate | aHR | 95% CI | 95% CI | *p* |
| --- | --- | --- | --- | --- | --- | --- | --- | --- |
| Without | 159,480 | 12,391 | 1,253,296.73 | 988.67 | Reference |  |  |  |
| With | 39,870 | 6,132 | 313,309.27 | 1,957.17 | 2.204 | 1.663 | 2.785 | < 0.001 |
| Driver of motor vehicle | 31,218 | 4,758 | 245,319.52 | 1,939.51 | 2.181 | 1.642 | 2.765 | < 0.001 |
| Passenger in motor vehicle | 1,490 | 230 | 11,708.16 | 1,964.44 | 2.212 | 1.670 | 2.794 | < 0.001 |
| Motorcyclist | 1,928 | 301 | 15,150.73 | 1,986.70 | 2.237 | 1.689 | 2.823 | < 0.001 |
| Passenger on motorcycle | 781 | 123 | 6,137.14 | 2,004.19 | 2.259 | 1.705 | 2.856 | < 0.001 |
| Pedal cyclist | 1,740 | 285 | 13,673.59 | 2,084.31 | 2.348 | 1.771 | 2.968 | < 0.001 |
| Pedestrian | 2,334 | 397 | 18,341.28 | 2,164.52 | 2.446 | 1.838 | 3.011 | < 0.001 |
| Others | 379 | 38 | 2,978.85 | 1,275.66 | 1.430 | 1.083 | 1.819 | 0.009 |

PYs = Person-years, Rate: per 100,000 PYs, aHR = Adjusted Hazard ratio: Adjusted for the variables listed in Table S4, CI = confidence interval
