# Supplementary material for: The Risk of Road Traffic Injuries Caused Hospitalization and the Risk of Mental Health Illness: A Nationwide, Matched‐Cohort, Population‐Based Study in Taiwan
Source: Brain Behav. 2025 Nov 10;15(11):e70993. doi: 10.1002/brb3.70993 (PMC12602460; doi:10.1002/brb3.70993)
Supplement: Supplementary file 6 — Table S6 Sensitivity analysis for factors of mental health illness subgroups by using Cox regression and Bonferroni correction for multiple comparisons [file BRB3-15-e70993-s002.doc]

**Table S6**. Sensitivity analysis for factors of mental health illness subgroups by using Cox regression and Bonferroni correction for multiple comparisons

|  | RTI inpatient | With (n = 39,870) | | | Without (n = 159,480) | | | With vs. Without (Reference) | | | |
| --- | --- | --- | --- | --- | --- | --- | --- | --- | --- | --- | --- |
| Sensitivity analysis | Mental health illness subgroups | Events | PYs | Rate | Events | PYs | Rate | aHR | 95%CI | 95%CI | *p* |
| Overall | Overall | 6,132 | 313,309.27 | 1,957.17 | 12,391 | 1,253,296.73 | 988.67 | 2.204 | 1.663 | 2.785 | < 0.001 |
|  | Anxiety | 1,423 | 313,309.27 | 454.18 | 2,589 | 1,253,296.73 | 206.58 | 2.359 | 1.780 | 2.981 | < 0.001 |
|  | Depression | 1,781 | 313,309.27 | 568.45 | 3,014 | 1,253,296.73 | 240.49 | 2.530 | 1.909 | 3.197 | < 0.001 |
|  | Bipolar disorder | 758 | 313,309.27 | 241.93 | 1,548 | 1,253,296.73 | 123.51 | 2.094 | 1.580 | 2.647 | < 0.001 |
|  | Sleep disorders | 1,132 | 313,309.27 | 361.30 | 2,331 | 1,253,296.73 | 185.99 | 2.098 | 1.583 | 2.650 | < 0.001 |
|  | ASD / PTSD | 54 | 313,309.27 | 17.24 | 13 | 1,253,296.73 | 1.04 | 5.237 | 3.965 | 6.612 | < 0.001 |
|  | Substance use disorders | 767 | 313,309.27 | 244.81 | 2,256 | 1,253,296.73 | 180.01 | 1.692 | 1.281 | 2.145 | < 0.001 |
|  | Dementia | 152 | 313,309.27 | 48.51 | 451 | 1,253,296.73 | 35.99 | 1.332 | 1.008 | 1.689 | 0.046 |
|  | Schizophrenia | 45 | 313,309.27 | 14.36 | 128 | 1,253,296.73 | 10.21 | 1.611 | 1.210 | 2.036 | < 0.001 |
|  | Personality disorders | 10 | 313,309.27 | 3.19 | 30 | 1,253,296.73 | 2.39 | 1.432 | 0.986 | 1.803 | 0.065 |
|  | Behavioral disorders | 10 | 313,309.27 | 3.19 | 31 | 1,253,296.73 | 2.47 | 1.303 | 0.971 | 1.794 | 0.079 |
| Events in the first year excluded | Overall | 5,579 | 293,727.44 | 1,899.38 | 10,579 | 1,174,965.68 | 900.37 | 2.096 | 1.482 | 2.577 | < 0.001 |
|  | Anxiety | 1,334 | 293,727.44 | 454.16 | 2,403 | 1,174,965.68 | 204.52 | 2.216 | 1.565 | 2.724 | < 0.001 |
|  | Depression | 1,678 | 293,727.44 | 571.28 | 2,834 | 1,174,965.68 | 241.20 | 2.354 | 1.666 | 2.897 | < 0.001 |
|  | Bipolar disorder | 725 | 293,727.44 | 246.83 | 1,442 | 1,174,965.68 | 122.73 | 1.971 | 1.392 | 2.425 | < 0.001 |
|  | Sleep disorders | 1,063 | 293,727.44 | 361.90 | 2,120 | 1,174,965.68 | 180.43 | 2.006 | 1.418 | 2.465 | < 0.001 |
|  | ASD / PTSD | 5 | 293,727.44 | 1.70 | 5 | 1,174,965.68 | 0.43 | 1.334 | 0.942 | 1.639 | 0.084 |
|  | Substance use disorders | 594 | 293,727.44 | 202.23 | 1,218 | 1,174,965.68 | 103.66 | 1.942 | 1.371 | 2.390 | < 0.001 |
|  | Dementia | 121 | 293,727.44 | 41.19 | 387 | 1,174,965.68 | 32.94 | 1.226 | 0.867 | 1.505 | 0.148 |
|  | Schizophrenia | 44 | 293,727.44 | 14.98 | 123 | 1,174,965.68 | 10.47 | 1.493 | 1.055 | 1.832 | 0.023 |
|  | Personality disorders | 8 | 293,727.44 | 2.72 | 22 | 1,174,965.68 | 1.87 | 1.328 | 0.936 | 1.630 | 0.102 |
|  | Behavioral disorders | 7 | 293,727.44 | 2.38 | 25 | 1,174,965.68 | 2.13 | 1.301 | 0.954 | 1.602 | 0.097 |
| Events in the first 5 years excluded | Overall | 3,912 | 181,389.58 | 2,156.68 | 7,615 | 725,592.84 | 1,049.49 | 1.842 | 1.196 | 2.278 | < 0.001 |
|  | Anxiety | 951 | 181,389.58 | 524.29 | 1,661 | 725,592.84 | 228.92 | 2.024 | 1.314 | 2.501 | < 0.001 |
|  | Depression | 1,237 | 181,389.58 | 681.96 | 2,093 | 725,592.84 | 288.45 | 2.105 | 1.368 | 2.604 | < 0.001 |
|  | Bipolar disorder | 518 | 181,389.58 | 285.57 | 1,067 | 725,592.84 | 147.05 | 1.707 | 1.099 | 2.115 | 0.001 |
|  | Sleep disorders | 753 | 181,389.58 | 415.13 | 1,534 | 725,592.84 | 211.41 | 1.751 | 1.137 | 2.162 | < 0.001 |
|  | ASD / PTSD | 0 | 181,389.58 | 0.00 | 0 | 725,592.84 | 0.00 | - | - | - | - |
|  | Substance use disorders | 341 | 181,389.58 | 187.99 | 826 | 725,592.84 | 113.84 | 1.462 | 0.985 | 1.871 | 0.065 |
|  | Dementia | 99 | 181,389.58 | 54.58 | 339 | 725,592.84 | 46.72 | 1.106 | 0.713 | 1.365 | 0.297 |
|  | Schizophrenia | 10 | 181,389.58 | 5.51 | 90 | 725,592.84 | 12.40 | 1.183 | 0.768 | 1.463 | 0.232 |
|  | Personality disorders | 0 | 181,389.58 | 0.00 | 5 | 725,592.84 | 0.69 | 0.000 | - | - | 0.999 |
|  | Behavioral disorders | 3 | 181,389.58 | 1.65 | 0 | 725,592.84 | 0.00 | ∞ | - | - | 0.999 |

PYs = Person-years, Rate: per 100,000 PYs, aHR = Adjusted Hazard ratio: Adjusted for the variables listed in Table S3, CI = confidence interval
